# Learning Experiences of Baccalaureate Nursing Students in an Ideological and Political Education‐Integrated Ophthalmic Nursing Course: A Qualitative Study

**DOI:** 10.1002/nop2.70540

**Published:** 2026-04-10

**Authors:** Jing Yan, Hua Liu, Lishuo Gao, Shuang Li, Xiaoying Zang, Qing Zhang, Yanan Xu

**Affiliations:** ^1^ Tianjin Key Laboratory of Retinal Functions and Diseases, Tianjin Branch of National Clinical Research Center for Ocular Disease, Eye Institute and School of Optometry Tianjin Medical University Eye Hospital Tianjin People's Republic of China; ^2^ School of Nursing, Tianjin Medical University Tianjin People's Republic of China

**Keywords:** curriculum‐based ideological and political education, learning experience, ophthalmic nursing

## Abstract

**Aim:**

This study aimed to explore the learning experiences of baccalaureate nursing students in a Curriculum‐based Ideological and Political Education integrated Ophthalmic Nursing course, in order to provide evidence for the refinement of future pedagogical frameworks.

**Design:**

A descriptive phenomenological approach was adopted within a qualitative research design.

**Methods:**

Participants were recruited using purposive sampling from third‐year baccalaureate nursing students enrolled in the Ophthalmic Nursing course. Data were collected through in‐depth, semi‐structured individual interviews from 29 November to 13 December 2024. All interviews were audio‐recorded and transcribed verbatim. Data analysis followed Colaizzi's seven‐step method for phenomenological analysis.

**Results:**

Twelve individual interviews were completed. Analysis of the data revealed three main themes: (1) opinions on pedagogical approaches and interactive modalities; (2) a requirement for clinically focused educational content; and (3) appropriate Curriculum‐based Ideological and Political Education content, yet undiversified delivery formats.

**No Patient or Public Contribution:**

Participants were nursing students; no patients, service users, or members of the public were involved in the design, conduct, or reporting of this research.

## 1 Introduction

Professional nursing is playing an increasingly important role in baccalaureate nursing programmes in China. The National Health Commission of China issued the Action Plan for Further Improvement of Nursing Services (2023–2025), which proposes promoting the specialisation of nursing (National Administration of Traditional Chinese Medicine, National Health Commission of the People's Republic of China 2023). The 14th Five‐Year National Ophthalmic Health Plan (2021–2025) emphasised that eye health is a vital component of national well‐being (National Health Commission of the People's Republic of China 2022).

Visual impairment, including blindness, has become a social issue that seriously affects individuals' physical and mental health, as well as their quality of life, thereby increasing the burden on families and society. Tianjin Medical University therefore introduced a new elective course in ophthalmic nursing in 2022 and aims to cultivate baccalaureate nursing students' core knowledge and practical skills in ophthalmic care. The Ophthalmic Nursing course is offered during the second semester of the third academic year, strategically scheduled after students have acquired foundational knowledge. The course is taught by three senior ophthalmic specialist nurses, all holding Master's degrees, who are officially employed as part‐time lecturers from Tianjin Medical University Eye Hospital.

According to the Teaching Guidelines for Curriculum‐based Ideological and Political Education in Nursing Professional Courses (hereinafter referred to as the Guidelines), the Ophthalmic Nursing course is required to integrate Curriculum‐based Ideological and Political Education (CBIPE) objectives into its syllabus (An et al. 2023). Teaching materials and course content for Ophthalmic Nursing at Tianjin Medical University are autonomously determined by educators based on instructional hours and disciplinary priorities. Over the past 3 years, its teaching outlines, pedagogical framework, and CBIPE design have been systematically compiled by faculty through synthesising multi‐source pedagogical materials and incorporating evidence from expert consensus statements (Grade A) and clinical practice guidelines (CPGs). The primary challenge in Ophthalmic Nursing lies in its limited 16‐credit‐hour curriculum, which necessitates the simultaneous integration of professional knowledge and CBIPE elements. To ensure teaching quality enhancement, a rigorous evaluation of the current pedagogical implementation is necessary. This study forms part of a college‐wide research project aimed at improving the pedagogical framework design of the CBIPE‐integrated Ophthalmic Nursing course. The main contribution of this study is to explore baccalaureate nursing students' learning experiences with the Ophthalmic Nursing course, to elucidate the intrinsic significance embedded in observed phenomena, and to provide evidence for the further refinement of curricular pedagogical frameworks.

## 2 Background

The concept of CBIPE was first clearly introduced by Xi Jinping, President of the People's Republic of China, at the National Conference on Ideological and Political Work in Colleges and Universities in 2016 (Wu and Hu 2016). CBIPE refers to an educational paradigm that integrates socialist core values, ethical principles, and political awareness into disciplinary instruction through implicit curriculum design. The Teaching Steering Committee for Nursing Programs in Higher Education Institutions (under the Ministry of Education) formulated guidelines that specify the objectives of CBIPE for nursing education across five dimensions: (1) strengthening professional conviction, (2) enhancing national cohesion, (3) advancing legal literacy, (4) reinforcing scientific rigour, and (5) improving professionalism (An et al. 2023). Moreover, implementing CBIPE in professional nursing courses is not only a national policy but also an urgent need within the nursing profession today.

A global nursing shortage has emerged as a critical challenge (WHO 2020), with evidence from China showing persistently high turnover intentions among clinical nurses. Zhang et al. (2017) found that professional identity was one of the key factors leading newly graduated nurses to leave the profession, highlighting the importance of supporting the development of professional identity among new nurses. Dai et al. (2022) reported that baccalaureate nursing programs incorporating CBIPE significantly enhance nursing students' sense of professional identity. Globally, numerous studies have also explored innovative pedagogical practices in baccalaureate nursing education to foster professional identity (Simmonds et al. 2020).

An increasing number of nursing educators in China have begun to develop CBIPE frameworks within nursing courses. A systematic review summarised that current studies on the development of CBIPE have covered all academic levels of nursing, including higher vocational, baccalaureate, and postgraduate education, with 18 courses mentioned in published articles (Liu et al. 2023). A scoping review identified 19 studies covering 11 courses related to CBIPE design in professional nursing education (e.g., Community Health Nursing 3, Internal Medicine Nursing 3, Nursing Management 3), none of which involved Ophthalmic Nursing. These studies primarily utilised quantitative evaluation indicators such as standardised scales, self‐designed questionnaires, and academic performance metrics, with no exploration of students' subjective experiences through qualitative methods (Yang et al. 2022).

Globally, while the concept of CBIPE has not been explicitly proposed in other countries, ideological and political education in nursing is integrated into broader pedagogical frameworks, including ethics education, national education, moral education, and civic literacy programmes (Liang and Wen 2023). Another systematic review by Shadi et al. (2024) reported that moral sensitivity in nursing students improves through specialised ethical knowledge and presence in educational and professional settings, and that this moral sensitivity enhances the quality of nursing care and the effective management of ethical challenges (2024).

Under these circumstances, Ophthalmic Nursing educators at Tianjin Medical University intend to develop effective and feasible pedagogical frameworks, which necessitate a systematic evaluation of current learning outcomes. CBIPE evaluation index systems for baccalaureate nursing courses were developed by both Mamengwei et al. (2022) and Yan et al. (2023). Teaching content, teaching methods, and students' cognition are three of the highest‐weighted factors in both evaluation index systems, particularly requiring students' experiences as a form of subjective evaluation.

## 3 Methods

### 3.1 Aims, Research Questions, and Objectives

This study aimed to explore baccalaureate Nursing Students' experiences in an ophthalmic nursing course integrated with Curriculum‐based Ideological and Political Education. The purpose was to evaluate the teaching process and gather insights to inform future pedagogical reforms.

The decision to adopt a qualitative design was informed by Moorley and Cathala's (2019) assertion that such methods are essential for elucidating nuanced subjective experiences—in this case, students' perceptions of integrating ideological‐political education into clinical learning. These deeply personal phenomena resist quantitative measurement, yet they are vital for developing transformative curricula.

### 3.2 Design

This study employed a descriptive phenomenological approach within qualitative research to systematically document the lived experiences of third‐year nursing students. Drawing on Moran's (2002) interpretation of Husserlian phenomenology, this study sought to remain free from unexamined presuppositions and achieve a “maximum intuitive presentation” of the experience (Matua and Van Der Wal 2015). To operationalise these principles, we followed Colaizzi's seven‐step methodology, which included: (a) maintaining phenomenological reduction via rigorous bracketing; (b) applying eidetic reduction to distil essential meanings; and (c) achieving intersubjective validation through member checking (Morrow et al. 2015).

Data were collected through semi‐structured interviews, conducted in accordance with Kallio et al.'s (2016) five‐phase guide. As the principal method for obtaining rich descriptions in phenomenology (Jackson et al. 2018), one‐to‐one interviews allowed us to critically examine experiences that are often taken for granted, revealing their hidden meanings and essences.

During the in‐depth semi‐structured interviews, the interviewer outlined the interview process, introduced the discussion topic, and explained the interview tasks to the participants. The interview outline was initially drafted by the research team through an initial literature review and iterative consultations with three qualitative research experts, including two clinical ophthalmic nursing specialists and a nursing education curriculum expert. Consultants provided written consent for their contributions to be acknowledged. The interview outline was pre‐tested in two phases: first with one domain expert to assess academic rigour, followed by two students to evaluate participant comprehension; these students were excluded from the final analysis to avoid pre‐test bias. The final outline comprised six questions. In addition, the researchers obtained participants' consent to record the interviews to ensure comprehensive data collection (Morgan et al. 1998).

The semi‐structured interviews were conducted by the first author (a female nurse‐in‐charge at Tianjin Medical University Eye Hospital with 17 years of ophthalmic nursing experience), who concurrently served as principal investigator of the institutional research project. Notably, she held no teaching roles in Ophthalmic Nursing. The interviewer had received training in conducting phenomenological qualitative research. Furthermore, the interviews were conducted after class hours and did not affect participants' course assessments, thereby protecting their rights and ensuring transcript anonymity.

### 3.3 Participants

A guide to phenomenological research suggests that the number of participants commonly ranges from 6 to 20 (Wilson 2015). Of the 43 students enrolled in the 2024 Ophthalmic Nursing course, initial recruitment via WeChat (China's dominant messaging platform) exclusively targeted individuals with complete attendance records. Among respondents expressing interest, judgement sampling was employed according to Shorten and Moorley's (2014) guide, based on two criteria: (a) Meeting predefined eligibility criteria and possessing lived experience relevant to research questions. (b) Ensuring sample credibility through participant diversity across gender, geographical backgrounds (Northeast, Northwest and Southern China), and career decision‐making pathways. Data collection ceased upon reaching saturation, yielding a final sample of 12 participants with no attrition.

### 3.4 Data Collection

Data were collected through individual, face‐to‐face, in‐depth semi‐structured interviews with 12 participants. Interviews were conducted in a quiet classroom, each lasting 25–40 min, without repeated sessions, between 29 November and 13 December 2024. The interview began with an introductory stage explaining CBIPE, followed by a transition from general to specific questions addressing the research objective. In addition to verbal responses, interview notes also captured participants' nonverbal behaviours and emotional expressions. The interviews were recorded using an iFLYTEK digital recorder. The audio recordings were transcribed using the iFLYTEK Hear app (developed by iFLYTEK Co. Ltd., China), and subsequently checked by the first author, who reviewed the text while listening to the recordings. Transcription was completed within 48 h of each interview. Finally, the transcripts were reviewed by another member of the research team against the original recordings. Recruitment of additional participants was deemed unnecessary, as data saturation was reached after 12 interviews.

The semi‐structured interview schedule, including the key prompts and follow‐up questions, is summarised in Table 1.

**TABLE 1:** Interview outline.

No.	Questions to guide the participants
1.	Could you describe your learning experience regarding the teaching objectives and content design of the Ophthalmic Nursing course?
2.	What were the main barriers to your engagement with this course, and what strategies proved effective in overcoming them?
3.	Can you reflect on your learning experiences with the different teaching methods used (e.g., PPT presentations, instructional videos, manikin‐based practice sessions, case analysis, interactive elements)? Could you share an example of a class where the method significantly helped you understand the content?
4.	Do you perceive this course as beneficial for your future nursing career? If yes, which aspects (e.g., technical procedures, theoretical knowledge, ethical decision‐making) do you consider most transferable?
5.	Could you express your perceptions regarding the integration approaches of Ideological and Political Education elements within the Ophthalmic Nursing course?
6.	Could you elaborate on how the course's ideological and political elements impacted your development of nursing professional identity and ethical practice competencies? Could you share examples?

### 3.5 Data Analysis

The data were analysed following Colaizzi's (1978) seven‐step procedure (Table 2). The first author began by immersing herself in the data through multiple, iterative readings of all interview transcripts. Significant statements directly pertaining to the phenomenon under investigation were then extracted. During this phase, any ambiguous expressions or those lacking clear phenomenological relevance were deliberately set aside to preserve analytical focus; a total of 172 verbatim statements were retained for further analysis.

**TABLE 2:** Steps in Colaizzi's descriptive phenomenological method.

Step	Description
(1) Familiarisation	The first author immersed herself in the verbatim interview transcripts, conducting iterative readings of all participants' narrative records.
(2) Identifying significant statements	All interview transcripts were systematically coded using NVivo 15. We extracted 172 verbatim phrases (e.g., “*I think case studies are an attractive teaching method*”), performed line‐by‐line coding, and excluded ambiguous metaphors.
(3) Formulating meanings	Latent meanings were interpreted (e.g., transforming “Not knowing each other well makes it awkward to speak up in group work” into “the group format inhibited effective collaboration”). These interpretations were grouped into 54 initial thematic clusters.
(4) Clustering themes	The data were systematically partitioned, labelled, and categorised. Synthesised clusters into 18 thematic groups (e.g., “a strong desire for clinical practice opportunities”).
(5) Developing an exhaustive description	A 9‐page phenomenological account was drafted, providing a comprehensive description of the phenomenon that incorporated all themes generated in Step 4.
(6) Producing the fundamental structure	The exhaustive description was distilled to its fundamental structure (e.g., “arrange extracurricular practice to enhance the effectiveness”).
(7) Seeking verification of the fundamental structure	The fundamental structure was returned to a subset of participants for member‐checking; no revisions to prior analytical steps were proposed.

Using NVivo 15, the extracted statements were coded. Through iterative reflection and memo‐writing, these codes were developed into formulated meanings, moving beyond the surface level of the transcripts to capture latent aspects of participants' experiences. These interpretations were initially organised into 54 thematic clusters. A thematic clustering approach was subsequently adopted, wherein related meanings were progressively grouped into 18 thematic groups. To enhance analytical rigour, this clustering process was undertaken independently by two researchers; any discrepancies that arose were resolved through discussion and mutual agreement.

All identified themes were synthesised into a nine‐page exhaustive description of the phenomenon. This comprehensive account was then distilled to produce a concise fundamental structure capturing the essence of the experience. To ensure credibility and intersubjective validation, this fundamental structure was returned to a subset of participants for member checking; all who provided feedback confirmed that the findings resonated with their lived experiences, and no amendments to the analytical structure were requested. The reporting of this study adhered to the Consolidated Criteria for Reporting Qualitative Research (COREQ), a 32‐item checklist.

### 3.6 Ethical Considerations

The study was approved by the Ethics Committee of the Tianjin Medical University Eye Hospital (approval number: 2024KY‐38) and was conducted in accordance with the ethical standards of the Declaration of Helsinki (2024 version) (World Medical Association 2025). All participants provided written informed consent which had been reviewed and approved by the Ethics Committee.

## 4 Results

This study included 12 students from the Tianjin Medical University (Table 3). The mean age of participants was 21 years. The sample comprised four males (33.3%) and eight females (66.7%), with eight from Northeast China (66.7%), three from Southeast China (25%), and one from Northwest China (8.3%). Three participants (25%) reported that their choice of nursing was not self‐selected, having been influenced by external factors such as family expectations or through academic programme adjustments, meaning they were transferred to non‐preferred majors via the institutional allocation system—a common practice in Chinese higher education used to balance programme capacities. Analysis of the interview data resulted in the extraction of 172 effective semantic reference points which were coded into 54 preliminary categories reflecting the complexity of participants' perspectives on their learning experiences. Following thematic analysis, students' perceptions of the CBIPE‐integrated Ophthalmic Nursing course were categorised into 18 thematic codes that coalesced into three major themes and six sub‐themes. The three themes were related to aspects of teaching approach, course content and arrangement, and CBIPE integrating, and were defined as follows: (1) opinions on pedagogical approaches and interactive modalities; (2) requirement for clinically‐focused educational content; (3) CBIPE content appropriate yet delivery formats undiversified (Table 4).

**TABLE 3:** Characteristics of interview participants (*n* = 12).

Series number	Gender	Age	Autonomy in major selection	Region
S1	Female	21	Self‐selected	Tianjin, China (Northeast)
S2	Male	22	Not self‐selected	Hebei, China (Northeast)
S3	Male	22	Self‐selected	Shandong, China (Northeast)
S4	Female	21	Self‐selected	Shandong, China (Northeast)
S5	Female	21	Self‐selected	Xinjiang, China (Northwest)
S6	Male	21	Self‐selected	Jiangsu, China (Southeast)
S7	Female	20	Self‐selected	Zhejiang, China (Southeast)
S8	Male	21	Self‐selected	Tianjin, China (Northeast)
S9	Female	21	Not self‐selected	Tianjin, China (Northeast)
S10	Female	20	Not self‐selected	Tianjin, China (Northeast)
S11	Female	22	Self‐selected	Hebei, China (Northeast)
S12	Female	21	Self‐selected	Jiangsu, China (Southeast)

**TABLE 4:** Themes and sub‐themes.

Themes	Sub‐themes	Codes
Opinions on pedagogical approaches and interactive modalities	Opinions on pedagogical approaches	Have a deep impression of the use of case studies
		Appreciation for video‐based and animated multimedia materials
	Limited engagement in interactive activities	Recognise that interaction could enhance engagement
		Feel awkward during interactive sessions
		The conventional roll call‐based questioning method is outdated
		Appreciate receiving encouragement after answering questions
Requirement for clinically‐focused educational content	Preference for disease‐based chapters over anatomy	The anatomy section is monotonous
		Like to learn knowledge based on eye diseases
	Preference for operational content in nursing education	Nursing operational skills are important
		Opinions on equipment‐based procedures
CBIPE content appropriate yet delivery formats undiversified	Appropriateness of ideological and political integration	CBIPE elements were meaningfully integrated with course content
		Medical innovations' contributions to human welfare
		Improving students' awareness of professional compliance
	CBIPE delivery formats undiversified	Limitations in CBIPE pedagogical design: predominantly relied on PowerPoint‐based delivery
		“Corneal Commemoration and Educational Activity” was particularly impressive
		Disappointment at the lack of opportunities to hear directly from role models
		A strong desire for clinical practice opportunities
		Particular interest in contemporary news stories

### 4.1 Theme 1: Opinions on Pedagogical Approaches and Interactive Modalities

#### 4.1.1 Sub‐Theme 1: Opinions on Pedagogical Approaches

Half of the participants (6/12) reported that they were particularly impressed by the use of case studies. They found these helpful in demonstrating nursing procedures, with one participant specifically highlighting the case involving an emergency eye condition. However, some noted that the case presentations were overly literal, requiring students to visualise real‐world scenarios independently.

It was helpful to include some case studies—like a specific individual with a certain condition. This made the content more practical and relatable. (S6, age 21)

I think case studies were an attractive teaching method. Cases tied to specific chapters really sparked my interest and made the content more engaging. However, we didn't see any case‐related videos. (S11, age 22)

Three participants (25%) expressed appreciation for video‐based and animated multimedia materials, although these were used less frequently than PowerPoint presentations.

I think it was beneficial to incorporate knowledge about animation, such as its principles and mechanisms. Using graphics or animations made it easier to understand and visualise complex concepts. (S1, age 21)

The teachers' PPT slides were well‐designed, with excellent integration of text and images. It might also be beneficial to add more videos to further enhance the learning experience. (S6, age 21)

#### 4.1.2 Sub‐Theme 2: Limited Engagement in Interactive Activities

Although students recognised that interaction could enhance engagement, many still exhibited reluctance to participate voluntarily in classroom activities. Half of the participants (6/12) described feeling ‘awkward’ during interactive sessions, particularly group discussions, as they were placed with peers from other classes whom they did not know well. Two of these students further noted that such groupings led to inefficient discussions.

I agreed that it was beneficial to include a few practice questions during each session to summarise key points, but I still feel too shy to speak up in class when everyone turns to look at me. (S5, age 21)

I think I'm more likely to participate when the topic interests me or when I already understand the content. Since it's an elective, and group members come from different classes, discussions often felt awkward and disconnected—even though we had a WeChat group. (S6, age 21)

The main issue is unfamiliarity among group members. Some people just don't engage, and that makes the discussion less effective. (S8, age 21)

Because most of us don't know each other well, group discussions often remain superficial and lack depth. The group format wasn't as effective as it could be. (S10, age 20)

In addition, five participants (41.7%) considered the conventional roll‐call‐based questioning method outdated and ineffective. Four participants (33.3%) suggested that the use of digital tools such as random selection apps could improve fairness and engagement. One student proposed a peer‐nomination mechanism to reduce anxiety and foster a more comfortable environment.

I agree that forcing students to respond is tough, especially when enthusiasm is low. A random selection tool might encourage participation—even if it feels slightly forced. (S2, age 22)

To promote active participation, I think a random selection function—like that on the Chaoxing platform—could keep students more alert and engaged. (S7, age 20)

It's easier to interact with classmates I'm familiar with. The roll‐call method made me anxious. I'd prefer being nominated by a peer—it might even be fun and reduce pressure. (S10, age 20)

Moreover, four participants (33.3%) appreciated verbal encouragement or small rewards after answering questions. They suggested that such reinforcement—either through small gifts or teacher praise—enhanced engagement. However, they also expressed disappointment that class participation was not rewarded with additional credit.

During one session, the teacher gave out small gifts like notebooks. It really worked—more students participated. But we weren't given extra marks for contributing, which was disappointing. Encouragement is also important—we need emotional support, not just academic credit. (S1, age 21)

Incentives like small gifts increased student enthusiasm. Even though the gifts were limited to the first class, motivation remained high in the following sessions once students realised they could actively contribute. (S4, age 21)

### 4.2 Theme 2: Requirement for Clinically‐Focused Educational Content

#### 4.2.1 Sub‐Theme 1: Preference for Disease‐Based Chapters Over Anatomy

Seven participants (58.3%) described the anatomy section of the course as monotonous. In contrast, eight participants (66.7%) expressed a clear preference for chapters focused on eye diseases—such as cataract, myopia, glaucoma, and retinal disorders—which they considered to be more relevant to clinical practice. Specifically, four participants referred to myopia, three to glaucoma, and one to retinal disease.

What really grabbed me in Ophthalmic Nursing were the chapters on glaucoma and myopia. My family has a glaucoma patient, and I have myopia myself. Learning these chapters helped me understand daily care methods for both me and my family. (S1, age 21)

The content that really caught my attention was about myopia and astigmatism. Since I actually have these conditions myself, I paid extra close attention during those lectures because I really wanted to understand them more deeply. (S11, age 22)

I liked the chapter on glaucoma nursing care. During my clinical rotations, I often encountered patients with a history of glaucoma. That experience motivated me to explore evidence‐based nursing interventions, which heightened my interest in the topic. (S5, age 21)

Seven of the 12 students (58.3%) felt the anatomy section was monotonous, while eight of twelve students (66.7%) expressed that they like to learn knowledge based on eye diseases such as cataract, myopia, glaucoma, and retinal diseases because they felt those were more useful for clinical work. Four of them specifically referred to myopia, three of them referred to glaucoma, and one student mentioned retinal disease.

What really grabbed me in Ophthalmic Nursing were the chapters on glaucoma and myopia. My family has a glaucoma patient, and I have myopia myself. Learning these chapters helped me understand daily care methods for both me and my family. (S1, 21 years of age)

The course content that really grabbed my attention was about myopia and astigmatism. Since I actually have these issues myself, I paid extra close attention during those lectures because I really wanted to dig deeper and understand more about them. (S11, 22 years of age)

I like the chapter on glaucoma nursing care. During my clinical rotations, I encountered individuals with a history of glaucoma frequently. This experience motivated me to delve deeper into evidence‐based nursing interventions for the patient's needs, which naturally heightened my interest in this field. (S5, 21 years of age)

#### 4.2.2 Sub‐Theme 2: Preference for Operational Content in Nursing Education

Three‐quarters of the participants (9/12) identified operational nursing skills as a particularly important aspect of the course. One chapter focused specifically on these skills. During this session, the instructor brought a manikin head and relevant materials to facilitate hands‐on practice. Several students were invited to the front of the classroom to demonstrate the procedures.

On the one hand, students expressed strong interest in practice‐based teaching and found in‐class hands‐on activities highly effective.

I distinctly recall one session when the teacher brought in a manikin head model for practising eye drop administration. This procedure, which I understand is routine in ophthalmic nursing, struck me as directly relevant to clinical practice. (S11, age 22)

During my neurology rotation, I cared for patients who required eye drop administration and conjunctival sac irrigation. This reminded me of our ophthalmic nursing lectures, where the teacher used a manikin head to demonstrate techniques. We were encouraged to practise these skills with hands‐on feedback, which helped bridge theory with clinical competence. The ophthalmic examination procedures using equipment also demonstrated clear clinical relevance. (S12, age 21)

On the other hand, participants held differing views regarding equipment‐based procedures. Some considered medical equipment to be an integral part of operational skills, while others viewed it as overly specialised and difficult to comprehend.

I didn't feel very familiar with the equipment—perhaps it's too specialised. It was difficult to study those topics in depth. (S10, age 20)

Several participants also felt that other chapters in the course lacked sufficient opportunities for practical engagement.

### 4.3 Theme 3: CBIPE Content Appropriate Yet Delivery Formats Undiversified

#### 4.3.1 Sub‐Theme 1: Appropriateness of Ideological and Political Integration

All participants reported that the ideological and political components of the Ophthalmic Nursing course left a strong impression. They felt that the CBIPE elements were meaningfully integrated with course content and had a subtle yet positive influence on their values and professional attitudes.

Half of the students (6/12) cited the chapter on cataract surgery as a representative example. They noted that the inclusion of public welfare initiatives in cataract treatment highlighted the noble ethics of medical professionals. Some students also expressed appreciation for how innovations in cataract microsurgery showcased the contributions of Chinese medical science to global health. Moreover, the course's emphasis on standardised procedural checks and relevant legal frameworks was credited with improving students' awareness of professional compliance.

Topics like cataract surgery and perioperative care were very relatable because we encounter these kinds of individuals quite often in daily life. The ideological and political elements in that chapter were also easy to grasp. The invention of cataract surgery has brought many people back to light, and through the stories of medical teams, we saw the noble ethics of doctors. (S10, age 20)

Patients with cataracts could go blind if they don't receive treatment. Doctors from the eye hospital volunteer in border regions to provide public welfare cataract surgery. I think this kind of compassionate medical action really inspires us as students. It made us more empathetic and socially responsible. (S11, age 22)

The teacher shared how pioneering Chinese professors introduced microsurgical techniques for cataract treatment and established standardised training programmes. This content aligned perfectly with the theoretical aspects of the chapter and reinforced a sense of national pride in medical innovation. (S6, age 21)

One teacher used the example of eye trauma to illustrate the idea of ‘compassion in medicine.’ It offered valuable lessons on humanistic care. At the same time, we learned practical skills and the importance of following procedures like pre‐operative checklists, which deepened our understanding of professional standards. (S4, age 21)

#### 4.3.2 Sub‐Theme 2: CBIPE Delivery Formats Undiversified

Three‐quarters of the participants (9/12) identified limitations in the current pedagogical design of CBIPE, which predominantly relied on PowerPoint‐based delivery. However, students described the “Corneal Commemoration and Educational Activity” as particularly impressive. During the corneal nursing chapter, the teacher introduced this traditional event held by Tianjin Medical University Eye Hospital. Three participants (25%) volunteered to take part in the activity during the Qingming Festival (4 April 2024). They reported that the experience deepened their understanding of selfless devotion and strengthened their professional convictions.

The eyes are such a vital organ for everyone, and corneal transplantation is truly remarkable—it gives people who lost their sight a chance to see again. Through the course, I learned about real individuals who made this selfless contribution, which really moved me. I chose nursing three years ago because of my parents' will, but now I understand the professional convictions of nursing for myself. (S9, age 21)

During the Qingming Festival Corneal Commemoration and Educational Activity, I listened to the father of a child who received a corneal transplant read a thank‐you letter. Regaining sight completely transformed the patients' lives, and the families expressed their deep gratitude to the medical staff and donors. My classmates and I were deeply moved—it really highlighted the selfless spirit of the donors. (S4, age 21)

Some students expressed disappointment at the lack of opportunities to hear directly from role models involved in public welfare surgery or to observe clinical practice firsthand. Three participants (all male, 25%) expressed particular interest in contemporary news stories involving medical professionals overcoming complex challenges, which they felt helped strengthen their professional identity. One participant noted that the teacher's selected news materials were somewhat outdated.

I'd prefer to hear role models share their own experiences and stories—it helps us empathise and better understand their emotions. (S11, age 22)

I particularly appreciate how the course integrates real‐world clinical cases, especially trending news from the internet. For instance, the teacher discussed a keratitis patient who delayed seeking care and developed severe complications. These examples made the lessons more relevant and impactful, and encouraged us to strengthen our professional convictions. (S3, age 22)

In addition, five participants (41.6%) expressed a strong desire for clinical practice opportunities to better connect ideological and political education with real‐world healthcare contexts.

The ophthalmic nursing course did not include hospital visits to support CBIPE learning. I would like to visit hospital departments and observe nurse–patient communication—it would help me understand their real‐life conditions more clearly. (S5, age 21)

When it comes to humanistic care, I think firsthand experience is what truly makes it meaningful. (S7, age 20)

## 5 Discussion

The aim of this study was to describe baccalaureate nursing students' learning experiences with the Curriculum‐based Ideological and Political Education integrated Ophthalmic Nursing course and to provide evidence for the further refinement of curricular pedagogical frameworks.

This study provides insights into the learning experiences of baccalaureate nursing students enrolled in a newly introduced Ophthalmic Nursing course integrated with Curriculum‐Based Ideological and Political Education (CBIPE). Participants expressed strong interest in disease‐specific nursing knowledge and hands‐on clinical skills, while demonstrating relatively less engagement with theoretical content, such as anatomy and equipment‐related procedures. Students also reflected on the limited classroom interaction, attributing it to unfamiliarity among group members and the absence of engaging participation strategies. The integration of CBIPE elements was recognised by students and perceived as effective in strengthening professional convictions, advancing legal literacy, and enhancing vocational competencies. Notably, the interviews revealed a strong student preference for diversified CBIPE modalities, suggesting an unmet demand for more innovative and engaging pedagogical strategies.

### 5.1 Teaching Procedure of Ophthalmic Nursing Calls for Innovation

Although students express greater interest in disease‐specific nursing content, such as cataract and glaucoma care, real‐life case discussions, and nursing procedures, Ophthalmic Nursing, as a specialised course in advanced nursing education, still bears the responsibility of delivering relevant theoretical knowledge and aligning with the intended learning outcomes of the syllabus. The course currently follows a traditional teaching sequence: anatomy, key concepts, aetiology, pathogenesis, clinical manifestations, nursing protocols, and nursing procedures. As participants described the anatomy session as “monotonous”, this may indicate a loss of interest from the outset of the course. Therefore, educators should adapt their teaching methods to make this comprehensive knowledge more accessible and engaging, rather than eliminating it altogether.

The current teaching methods in Ophthalmic Nursing primarily rely on PowerPoint presentations, with limited use of Case‐Based Learning (CBL) and a single practical nursing skills session. CBL was incorporated into the teaching of nursing protocols and operational procedures. Shohani et al. (2023) demonstrated that, compared to traditional lecture‐based methods, implementing CBL in specialised nursing curricula significantly enhances learners' academic satisfaction and self‐efficacy. Our semi‐structured interviews further revealed that participants tended to prefer clinical scenario immersion and practical training. This finding aligns with Lave and Wenger's Situated Learning Theory (SLT) (1991). Lave and Wenger characterised learning as embedded in everyday activities, context, and culture; fundamentally social; often unintentional rather than deliberate; and progressive with respect to learners' participation (O'Brien and Battista 2020). Furthermore, Wang (2021) proposed an innovative framework for integrating SLT into ideological and political education in higher education institutions, aiming to enhance its relevance and appeal.

These findings suggest a pedagogical innovation framework that integrates Case‐Based Learning (CBL) with Situated Learning Theory (SLT), which could systematically enhance educational effectiveness. Peng's study introduced scenario‐based pedagogy into anatomical education, transforming traditional rote‐learning models by integrating contextualised learning modules with relatable real‐life scenarios. This approach demonstrated significant advantages, including enhanced learner engagement, a shift in students' traditional perceptions of anatomy, stimulation of critical thinking, and improved ability to analyse and solve practical problems (Peng et al. 2011). For example, in the context of cataract nursing, and drawing on SLT principles, the introductory section of the chapter could be redesigned using a real‐life case scenario:

Your neighbour, Aunt Li, reports that her vision is gradually becoming blurred and she can no longer clearly see her grandson's face. Assume you are a junior nurse in an eye hospital—how would you help her?

Furthermore, some participants felt that equipment‐related examinations in Ophthalmic Nursing were less relevant to nursing practice and difficult to understand. In fact, the delivery of ophthalmic services is highly dependent on equipment, ranging from a simple torchlight to highly sophisticated diagnostic and therapeutic devices (Patel et al. 2010). It is essential for ophthalmic medical staff to develop full proficiency in the use of medical equipment, in order to maximise its effectiveness and to facilitate the timely detection and treatment of patients' conditions (Zhang et al. 2019). Educators should address students' misconceptions, help them understand the importance of equipment‐related examinations, and improve teaching methods by incorporating more hands‐on opportunities with equipment to enhance engagement. Therefore, educators plan to redesign the course by incorporating practical tools such as the iCare rebound tonometer in classroom sessions, allowing students to practise measuring intraocular pressure (IOP) while explaining the clinical significance of IOP monitoring in glaucoma management.

### 5.2 Unsatisfactory Interactive Activities

In the Ophthalmic Nursing class, students exhibited limited engagement in interactive activities. Some participants acknowledged that the Q&A section could help maintain focus and reinforce learning, yet they were reluctant to raise their hands. These reasons were identified by educators through the interviews. Firstly, the students were from different classes within the same academic year, and many were unfamiliar with one another. As a result, they felt too shy to participate in discussions or respond to questions. Secondly, they had become accustomed to interacting online—through platforms such as WeChat groups—rather than face‐to‐face. This phenomenon is likely linked to long‐term online learning habits developed by this generation of students from secondary school through the early stages of university, particularly during the COVID‐19 pandemic (2020–2022). Peer communication is a key component of student engagement (Kahu and Nelson 2017), yet students with this background appear to have been adversely affected. Liu and Mo (2022) suggested that online learning mediates the relationship between reduced social interaction, perceived psychological harm, perceptions of social distancing, and regulatory compliance. The observed lack of social skills among this generation also aligns with Bandura's *Social Learning Theory (SLT)*, which posits that individuals acquire social skills through the observation and imitation of modelled behaviours (Bandura 1989). Specifically, in‐person classroom activities (e.g., group discussions, role‐play) serve as critical rehearsal platforms, enabling students to refine communication strategies through real‐time feedback and peer interaction.

To better engage students in face‐to‐face activities, educators should promote participation through acceptable and inclusive strategies. The Chaoxing Learning Platform, officially approved by the university, is a potentially valuable tool that is widely accepted by students. Chaoxing is a multifunctional educational platform that enables in‐class sign‐ins, random student selection, resource sharing, group discussions, homework submission, testing, and more. Cheng and Zhou (2024) reported that blended learning of the Labour Education course using Chaoxing improved the efficiency of discussion‐based classroom teaching. Liu (2024) redesigned Unit 5 of The New Horizon College English Reading and Writing Course 3 into a blended learning format with Chaoxing. This not only stimulated students' enthusiasm for learning but also enabled educators to enrich the course and enhance teaching effectiveness. Chaoxing could facilitate the random selection of students to answer questions or perform procedural demonstrations in Ophthalmic Nursing sessions. According to *SLT*, peer communication is critical to learning; thus, educators should continue to prioritise offline interaction in group discussions, even when incorporating Chaoxing. Educators could group students by class, provided the numbers are relatively balanced, to encourage participation. Alternatively, randomised group allocation via the Chaoxing platform could be used alongside ice‐breaking activities to reduce initial interpersonal barriers. In future teaching practice, educators should recognise that student characteristics may vary across generations. Therefore, it is essential to adjust teaching strategies in a timely manner based on classroom dynamics and student feedback.

### 5.3 Limitations in the Forms Through Which CBIPE Was Delivered

Some of the ideological and political elements that left a strong impression on students included the efforts of doctors and nurses working in remote areas, the contributions of medical advances to human well‐being, and corneal donation initiatives. Participants felt that the integration of ideological and political education with the Ophthalmic Nursing curriculum was generally appropriate. However, they also noted limitations in the forms through which CBIPE was delivered. They expressed a desire for more diverse CBIPE teaching methods, such as hospital visits to understand the patient journey, lectures delivered by role models, or participation in community‐based activities. Several students specifically mentioned the Corneal Commemoration and Educational Activity as a particularly meaningful experience.

The participants' learning preferences aligned with David Kolb's Experiential Learning Theory (ELT), proposed in 1984. The theory outlines a cyclical process comprising four phases: concrete experience (CE), reflective observation (RO), abstract conceptualisation (AC), and active experimentation (AE) (Kolb 1984). In medical education, ELT is frequently applied to learning experiences embedded within curriculum designs that bring learners into contact with others in specific roles and contexts (Yardley et al. 2012). Today, ELT is widely applied in nursing education, including tertiary programmes, in‐service training, and clinical patient education (Zeng and Zhu 2019). Gu et al. (2024) explored CBIPE frameworks in paediatrics. The researchers organised student participation in hospital‐based activities for children with tumours—including daily reading, crafts, painting, and birthday celebrations—which enriched the ward environment and provided emotional support. These experiences subtly instilled in students a sense of professional responsibility and mission. Findings from our qualitative study suggest that ELT may be a valuable framework for CBIPE innovation in Ophthalmic Nursing. Observing real patients in clinical settings could enhance students' empathy, while witnessing the impact of medical advancements or learning from role models may improve the effectiveness of ideological and political education.

## 6 Limitation of the Study

As Ophthalmic Nursing is an elective course, the participants may not fully represent all baccalaureate nursing students in the same year group. The data were collected from a single university, which was the only institution in the city offering this course. This study employed a qualitative design. As such, the sample size was relatively small, with only 12 participants, which may not fully capture the diversity of student perspectives. The limited communication skills observed among students may be attributed to the COVID‐19 pandemic, during which they undertook a large number of online courses. This characteristic may not apply to students from other generational cohorts.

## 7 Conclusions

Ophthalmic Nursing is a newly introduced elective course for baccalaureate nursing students. Educators reflected on the course content, teaching methods, and the design of CBIPE based on students' feedback obtained through semi‐structured interviews. Students expressed preferences regarding the course content, explained reasons for the low level of classroom engagement, and identified limitations in the current forms of CBIPE. Future pedagogical innovations could involve integrating Case‐Based Learning (CBL) with Situated Learning Theory (SLT) to enhance student interest in course content; incorporating the Chaoxing Learning Platform and ice‐breaking activities to rebuild social competencies and promote offline peer communication; and organising extracurricular practice to strengthen the effectiveness of ideological and political education. Additionally, these findings warrant further investigation, and teaching strategies should be dynamically adjusted in accordance with students' evolving needs and circumstances.

## Author Contributions

**Jing Yan:** conceptualization, methodology, formal analysis, writing – original draft, project administration. **Hua Liu:** writing – review and editing, supervision. **Lishuo Gao:** validation, resources, supervision. **Shuang Li:** validation. **Xiaoying Zang:** supervision. **Qing Zhang:** resources. **Yanan Xu:** investigation.

## Funding

This study was funded by the School of Nursing, Tianjin Medical University, through the Specialised Enhancement Programme for Clinical Teaching (Grant number: 2024LCZX03), and funded by Tianjin Key Medical Discipline Construction Project (TJYXZDXK‐3‐004A‐2). The funders had no role in the design of the study, data collection, analysis, interpretation, or manuscript preparation.

## Ethics Statement

Ethical approval was obtained from the Ethics Committee of Tianjin Medical Eye Hospital prior to participant recruitment (Approval number: 2024KY‐38).

## Conflicts of Interest

The authors declare no conflicts of interest.

## Data Availability

The data that support the findings of this study are available on request from the corresponding author. The data are not publicly available due to privacy or ethical restrictions.
